# Supplementary material for: Changes in the vitreous body after experimental vitreous hemorrhage in rabbit: An interdisciplinary study
Source: PLoS One. 2023 Feb 6;18(2):e0281165. doi: 10.1371/journal.pone.0281165 (PMC9901783; doi:10.1371/journal.pone.0281165)
Supplement: Attachment — Submitted filename: 2 Response to Reviewers.doc [file pone.0281165.s002.doc]

**Response letter**

**Dear Reviewers,**

On behalf of my co-authors, we are very grateful to you for giving us an opportunity to revise our manuscript. Meanwhile, we appreciate you very much for your positive and constructive comments and suggestions on our manuscript entitled “*Changes in the Vitreous Body after Experimental Vitreous Hemorrhage in Rabbit: An Interdisciplinary Study*” (**ID: PONE-D-22-22747**).

We have studied reviewers’ comments carefully and tried our best to revise and improve the manuscript according to the comments. The following are responses and revisions I have made on an item-by-item basis.

**Responds to the reviewers’ comments:**

**Reviewer #1:** Dear reviewer Ayhan Kanat,

1) *Similarity analyze reveals that the manuscript is similar to other article(without references) ata rate 35%. It should be less than 30%. There are to aricle which are similar at a rate 8%. One of them is unpublished PDF. Other is an article (doi: https://doi.org/10.1101/2020.07.09.20150136) but this article was not aslo cited.*

**Response**: We appreciate it very much for this excellent suggestion, and we have done it according to your comments. As for the large similarity, we have made substantial modifications in sections and sentences, and quoted the literature you mentioned above (doi: <https://doi.org/10.1101/2020.07.09.20150136>) as the first reference in this manuscript. We do hope that the original degree and readability have been substantially improved.

2) *The aim of authors is not clear in the introduction section, but they wrote in first sentence of abstract. It should be emphasize in the introduction section.*

**Response**: We have supplied the aim of this study to the manuscript in the introduction section, line 68-70.

3) *In order to understand the changes after vitrous hemorrhage, the authors performed experiments in a rabbit model by injection of blood or saline, as well as control animals that did not undergo, so a SHAM group was used, but the finding of this group was not given in the result section.*

**Response**: In our study, the eyes of rabbits were assigned into two groups: study group (right eyes) – full autologous blood injected, and control one (left eyes, evaluate possible adverse effects of ocular trauma or exogenous fluid) – injected with equal volumes of saline in the same manner (Methods/Animal model construction/Line 98-101). No blank control animals that did not undergo (as a SHAM group) were included and not required to justify our statements. However, the ultrastructure images of the control group (saline injected) were further supplemented. And we will be happy to edit the text further, based on helpful comments from you.

Special thanks to you for your thoughtful comments.

**Reviewer #2**

1. *The motivation of study require to add to Introduction and the implication for clinical application also need to provide in Discussion.*

**Response**: We have supplied the study motivation and clinical application values in the introduction and conclusion sections respectively.

2. *Line 36 of the article: “and then subsequently…”, the then should be dropped.*

*Line 85, “fixed” should be replaced with “cured”*

**Response:** We are very sorry for our negligence of the presentation and the above errors you mentioned have been rectified (Line 59 and 138). Thanks for your advice.

3. *The English writing needs to improve by professional editor.*

**Response**: We apologize for some imprecise language of this paper. We worked on it for a long time, while the repeated addition and removal of sentences led to labored readability. We have now worked on both language and readability and have also involved native English speakers for corrections. We do hope that the language level and flow have been substantially improved.

Thanks again to the hard work of you!

We look forward to receiving your reply.

Kind regards,

Pengcheng Zhang

zhangipengcheng@outlook.com

Corresponding author: Hong Yan

E-mail: yan2128ts@med.nwu.edu.cn
