# Supplementary material for: Changes in the vitreous body after experimental vitreous hemorrhage in rabbit: An interdisciplinary study
Source: PLoS One. 2023 Feb 6;18(2):e0281165. doi: 10.1371/journal.pone.0281165 (PMC9901783; doi:10.1371/journal.pone.0281165)
Supplement: Attachment — Submitted filename: 1 Response to Reviewers.doc [file pone.0281165.s003.doc]

**Response letter**

**Dear Reviewers,**

On behalf of my co-authors, we are very grateful to you for your positive and constructive comments and suggestions on our manuscript entitled “*Changes in the Vitreous Body after Experimental Vitreous Hemorrhage in Rabbit: An Interdisciplinary Study*” [PONE-D-22-22747R2] - [EMID:869453ce1fd5a818].

We have studied the comments carefully and tried our best to revise and improve the manuscript according to the comments. The following are responses and revisions we have made on an item-by-item basis.

**Responds to the reviewers’ comments:**

**Reviewer #1:** Dear professor Ayhan Kanat,

1) *The author agrre that a control group is not required to justify their statements. Is there any previous study that use this method? If here is, I recommend them to cite that study. If not, this situation should be noted in the limitation section. The ultrastructure images of the control group (saline injected) were supplemented. It is difficult to accept the saline injected animal as a control group animal.*

**Response**: The intervention used in this study was an intravitreal injection of autologous blood, to explore the changes of vitreous itself after hemorrhage. That is, the focus is on intravitreous hemorrhage, rather than intravitreous injection. Therefore, a blank control in this study was not selected, but saline intravitreal injection. Similar method was also used for comparison, which has been cited in the manuscript (Line 101, new citations 15 and 16). Of course, it would be better to include blank control as well. Hence, it is pointed out in the limitation section (Line 305-306) that needs to be improved. We appreciate it very much for this excellent suggestion, and we will be happy to edit the text further, based on helpful comments from you.

2) *As part of the central nervous system, the optic nerve is surrounded by a distensible subarachnoidal space, designated as the optic nerve sheath. As a consequence of this communication, CSF can transfer freely between the intracranial and intraorbital subarachnoid space. This communication may be the reason why papilledema be a common neuro-opthalmological exam finding of intraocular pressure elevation. In this study, after intravitreal injection of blood or saline , IOP did not fluctuate significantly. How can the authors explain this situation?*

**Response**: It is true that the optic nerve is sensitive to changes in intraocular pressure. Vitreous fluid injection also likely causes IOP elevation. Thus, in the “Animal model construction” section of this manuscript, we mentioned that after the injection, the “anterior chamber puncture” procedure (Line 104-105) is designed to soften the eyeball, precisely to avoid excessive fluctuations of intraocular pressure.

3) *I have a concern about the author’ suggestion about that the retina can dynamically maintain the blood flow rate constant. What do you think the authors about the vasospasm in the ophthalmic artery? The manuscript require minor revision.*

**Response**: Special thanks to you for this valuable feedback. The dynamic regulation of retina for blood flow rate exists under a certain IOP range in physiological conditions. Once decompensation occurs, vasospasm or lesions may occur, that retinal detachment is directly related to the degree of vasospasm in the ophthalmic artery. We have revised the relevant content (Line 225-227, newly cited reference 19).

**Reviewer #2:** Dear Reviewer,

Thank you for appreciating our efforts to address your constructive comments, which really helped us improve the manuscript.

Thank both reviewers for your time and positive feedback for the evaluation of the revision.

We look forward to receiving your reply.

Kind regards,

Pengcheng Zhang

zhangipengcheng@outlook.com

Corresponding author: Hong Yan

E-mail: yan2128ts@med.nwu.edu.cn
